# Development of an AAPH-Induced Oxidative Stress Model in Bovine Mammary Epithelial Cells and Investigation of Its Molecular Mechanisms

**DOI:** 10.3390/antiox15040460

**Published:** 2026-04-08

**Authors:** Yuanyuan Zhang, Daqing Wang, Jiahui Wu, Zhiwei Sun, Guifang Cao, Yong Zhang

**Affiliations:** 1 College of Veterinary Medicine, Inner Mongolia Agricultural University, Hohhot 010018, China; zyyworkaccount@163.com (Y.Z.);; 2 Animal Embryo and Developmental Engineering Key Laboratory of Higher Education, Institutions of Inner Mongolia Autonomous Region, Hohhot 010018, China; 3 Inner Mongolia Autonomous Region Key Laboratory of Basic Veterinary Medicine, Hohhot 010018, China; 4 Tongliao Institute of Agricultural and Animal Husbandry Sciences, Tongliao 028000, China; 5 College of Life Sciences, Inner Mongolia University, Hohhot 010021, China

**Keywords:** bovine mammary epithelial cells, oxidative stress, AAPH, transmission electron microscopy

## Abstract

Bovine mastitis is a multifactorial inflammatory disease primarily characterized by inflammatory cell infiltration and the destruction of mammary alveoli. It is a major cause of reduced milk yield and quality. The imbalance between antioxidant defenses and the generation of reactive oxygen species (ROS), which occurs due to the high metabolic activity of the mammary gland during the periparturient period, increases the incidence of mastitis. During early lactation, especially in high-yielding dairy cows, the massive synthesis and secretion of milk increase the energy demand of mammary tissue, leading to excessive ROS accumulation. This results in cell membrane disruption and, ultimately, antioxidant dysfunction in the mammary tissue. This study established an in vitro oxidative stress model by treating bovine mammary epithelial cells (BMECs) with 2,2′-azobis(2-amidinopropane) dihydrochloride (AAPH). The optimal concentration of 1000 μmol/L AAPH was determined using the CCK-8 assay. Model validation showed that, compared to the control group, ROS levels were significantly elevated (*p* < 0.001) and mitochondrial membrane potential was significantly decreased (*p* < 0.001) in the AAPH-treated group. Transmission electron microscopy (TEM) analysis revealed that AAPH treatment caused ultrastructural damage, including reduced microvilli, mitochondrial swelling, disappearance of cristae, and vacuolization. Mechanistic studies demonstrated that AAPH treatment significantly upregulated the mRNA and protein expression of *AMPK*, *HMOX-1*, *mTOR*, *NOS*, and *SOD* (*p* < 0.001), while significantly downregulating CYP1A1 expression (*p* < 0.001). Pretreatment with N-acetylcysteine (NAC) effectively alleviated the oxidative stress damage caused by AAPH. This study successfully established an in vitro AAPH-induced oxidative stress model in BMECs and revealed its molecular mechanism of cellular damage. The damage occurs through modulation of the AMPK/mTOR signaling pathway and the regulation of antioxidant-related gene expression.

## 1. Introduction

The dairy industry is fundamentally connected to essential human sustenance and well-being. However, it faces significant challenges due to bovine mastitis [1]. Oxidative stress occurs when the balance between oxidative markers and the antioxidant defense system is disrupted, leading to an excessive accumulation of ROS and the activation of antioxidant genes [2,3]. ROS play a dual role in the body: at low levels, they transmit signals and promote cell proliferation, but in excess, they cause cellular damage and apoptosis [4]. 2,2′-azobis(2-amidinopropane) dihydrochloride (AAPH) is a water- soluble free-radical initiator that decomposes at 37 °C in the presence of oxygen to generate carbon-centered radicals. These radicals are rapidly converted to peroxyl radicals [5], which then attack membrane lipids, initiating lipid peroxidation. This process depletes endogenous antioxidants such as GSH and SOD, leading to an increase in ROS, loss of membrane integrity, and ultimately, cell apoptosis. Because the rate of radical production is constant, and the resulting damage is dose- and time-dependent, AAPH is widely used to establish in vitro oxidative-stress models for evaluating the antioxidant activity of natural products or drugs [6].

Currently, oxidative stress models in BMECs mainly rely on induction by H_2_O_2_ or lipopolysaccharide (LPS) [7,8]. However, these models have limitations, including intense oxidative bursts and confounding inflammatory responses. The sustained and controllable radical-releasing property of AAPH may more accurately mimic the chronic oxidative stress conditions in the mammary glands of dairy cows during the periparturient period. Given that oxidative stress in vivo during the periparturient period is characterized by persistent, low-grade ROS production rather than an acute burst, the use of AAPH to establish an in vitro model offers a controlled system that recapitulates this chronic oxidative environment. This allows for the mechanistic dissection of cellular responses—such as signaling pathway activation and gene expression changes—that are otherwise difficult to isolate in vivo due to the complex interplay of metabolic, endocrine, and immune factors. Therefore, establishing such an in vitro model provides a reliable platform for elucidating the molecular mechanisms that underlie oxidative stress-induced mammary epithelial damage, which are expected to translate to the in vivo context. Furthermore, the AMPK/mTOR signaling pathway is a central regulator of cellular energy metabolism and oxidative stress responses. However, its regulatory mechanisms in mammary epithelial cells and its association with mastitis remain poorly understood [9,10].

In this study, an in vitro oxidative stress model was established in BMECs through exposure to AAPH. The success of the model was verified by measuring intracellular ROS levels and mitochondrial membrane potential. Ultrastructural changes in the mitochondria following AAPH treatment were visualized using transmission electron microscopy (TEM). The transcriptional and protein expression of genes related to oxidative stress were quantified through immunofluorescence, qPCR, and Western Blot. This study aims to elucidate the molecular mechanism by which AAPH regulates oxidative stress in BMECs, thereby providing insights for the prevention and treatment of bovine mastitis and identifying novel therapeutic targets.

## 2. Materials and Methods

### 2.1. Establishment of an Oxidative Stress Model in BMECs Induced by AAPH

#### 2.1.1. Recovery and Cultivation of BMECs

The BMECs used in this experiment were isolated, purified, and identified by our laboratory. Cryopreserved third-generation cells were retrieved from liquid nitrogen and immediately thawed by rapid shaking in a 37 °C water bath until completely melted. The cell suspension was transferred to a centrifuge tube containing complete medium, gently mixed by pipetting, and centrifuged at 1000 rpm for 5 min. The supernatant was discarded to remove the cryopreservation solution. Fresh complete medium was added to resuspend the cell pellet, and after adjusting the cell density, cells were seeded into 25 cm^2^ culture flasks at a concentration of 1 × 10^5^ cells/mL. The complete medium used was DMEM/F12 basal medium supplemented with 10% fetal bovine serum (FBS) and 2% penicillin-streptomycin double antibiotic solution. The culture flasks were incubated statically in a 37 °C incubator with 5% CO_2_, and the medium was replaced every 2 days. When cells reached approximately 80–90% confluence, they were digested with 0.25% trypsin, passaged, and expanded. The sixth-generation cells in good growth condition were used for subsequent experiments.

#### 2.1.2. Main Reagents

Fetal bovine serum (FBS) from Excell Biological (Suzhou, China). DMEM/F12 medium, reduced-serum medium, 0.25% trypsin, and antibiotics were obtained from Gibco (Big Island, NY, USA). The Multisource Total RNA Miniprep Kit was sourced from AXYGEN (Silicon Valley, CA, USA), and the total protein extraction kit was obtained from Sangon Biotech (Shanghai) Co., Ltd. (Shanghai, China). Reverse transcription and qPCR kits were supplied by Vazyme (Wuhan, China), and the BCA protein concentration assay kit and 5× loading buffer were sourced from Beyotime (Shanghai, China). The pre-stained protein marker was from Bio-Rad (Herculaneum, CA, USA), and BSA was purchased from Amresco (Silicon Valley, CA, USA). The SDS-PAGE gel preparation kit was from Solarbio (Beijing, China). Rabbit monoclonal antibodies against AMPK, CYP1A1, HMOX-1, mTOR, NOS, and SOD were sourced from Proteintech (Wuhan, China), while goat anti-rabbit IgG-HRP, goat anti-mouse IgG-HRP, and mouse anti-β-actin monoclonal antibody were obtained from Affinity Biosciences (Changzhou, China). AAPH, N-Acetylcysteine (NAC), the Cell Counting Kit-8, ROS Assay Kit, and JC-1 Mitochondrial Membrane Potential Assay Kit were purchased from MedChemExpress (MCE) (Monmouth Junction, NJ, USA).

#### 2.1.3. Main Instruments

The following instruments were used in this study: the Bio-Rad horizontal electrophoresis system (Bio-Rad, Herculaneum, CA, USA), PowerPac universal power supply (Herculaneum, CA, USA), CFX96 real-time quantitative PCR system (Beijing Saibaiao Technology, Beijing, China), and Trans-Blot transfer apparatus (Bio-Rad, Herculaneum, CA, USA); the Thermo Fisher Scientific VeritiPro thermal cycler (Thermo Fisher Scientific, Wilmington, DE, USA); the Syngene G:BOX gel documentation system (Cambridge, UK); the Hitachi CP100WX ultracentrifuge (Tokyo, Japan); the Thermo Scientific Forma CO_2_ incubator (Wilmington, DE, USA); the Olympus IX73 inverted fluorescence microscope (Tokyo, Japan); and the BioTek Synergy H1 multimode microplate reader (Winooski, VT, USA).

#### 2.1.4. Establishment of an Oxidative Stress Model in BMECs

To determine the appropriate concentration and duration of AAPH treatment for inducing oxidative stress in BMECs, this study evaluated cell viability using the CCK-8 assay and analyzed changes in the expression of oxidative stress-related genes by qPCR. First, the concentration of AAPH was optimized. BMECs in good growth condition were digested with trypsin to prepare a cell suspension, which was seeded into 96-well plates at a density of 1 × 10^4^ cells per well in a volume of 100 μL per well. The plates were incubated at 37 °C in a 5% CO_2_ incubator for 24 h to allow cell attachment. Once cells reached approximately 80% confluence, the old medium was aspirated. Experimental groups were set as follows: blank control (complete medium only, no cells), negative control (cells with complete medium, without AAPH treatment), and AAPH treatment groups at various concentrations. For treatment groups, 100 μL of AAPH solution diluted in complete medium was added to achieve final concentrations of 0.2, 0.4, 0.6, 0.8, 1.0, 50, 100, 150, 200, and 250 mM, with three replicate wells per concentration. After 24 h of culture, the supernatant was discarded, and 100 μL of serum-free medium containing 10% CCK-8 reagent (Med-ChemExpress (MCE)---Monmouth Junction, NJ, USA) was added to each well, followed by incubation in the dark for 2 h. The OD value at 450 nm was measured using a microplate reader. The inhibition rate of cell viability was calculated using the formula inhibition (%) = [(Ac − As)/(Ac − Ab)] × 100%, where As represents the absorbance of the treatment wells, Ac represents the absorbance of the negative control wells, and Ab represents the absorbance of the blank control wells. Based on the results, the concentration of AAPH yielding an inhibition rate above 20% was selected as the treatment concentration for subsequent experiments.

Based on the AAPH concentration selected above, the optimal treatment duration was further determined. BMECs were seeded into 6-well plates at a density of 2 × 10^5^ cells per well. After cells reached 80–90% confluence, they were divided into a negative control group and an AAPH treatment group. The treatment group received complete medium containing the selected concentration of AAPH and was cultured for 6, 12, 24, and 48 h. At each time point, a corresponding negative control group (medium change without AAPH) was included, with three replicate wells per group. At the designated time points, the medium was discarded, and cells were washed twice with ice-cold PBS, followed immediately by total RNA extraction. RNA purity and concentration were assessed by UV spectrophotometry, and cDNA was synthesized by reverse transcription. The mRNA expression levels of oxidative stress-related genes were detected by qPCR, with β-actin used as an internal control. Based on the expression trends of the target genes across different time points, the time point showing the most significant differences in oxidative stress-related gene expression was selected as the optimal AAPH treatment duration for subsequent mechanistic studies.

### 2.2. Changes in Oxidative Stress-Related Indicators in BMECs After the Addition of AAPH

#### 2.2.1. ROS Levels in BMECs

BMECs were plated in a 6-well plate the day before the assay, ensuring 50–70% confluence at the time of measurement. On the following day, the treatment group had the culture medium removed and replaced with fresh medium containing 1000 μmol/L AAPH. The cells were then incubated at 37 °C in the dark for 6 h. For the positive control group, the positive-control reagent supplied with the kit was added to the cells, and incubation continued at 37 °C in the dark for 30 min to enhance intracellular ROS levels. After treatment, the medium was removed from both groups, and the fluorescent probe supplied in the kit was applied to each well. The cells were incubated at 37 °C for 30 min, then rinsed three times with DPBS to remove any unincorporated probe. ROS levels were observed under a laser-scanning confocal microscope (Nikon, Tokyo, Japan) with an excitation wavelength of 488 nm, and fluorescence intensity was analyzed using ImageJ1.48 V.

#### 2.2.2. Mitochondrial Membrane Potential Levels in BMECs

BMECs were plated in a 6-well plate the day before the assay, ensuring 50–70% confluence at the time of measurement. On the following day, the treatment group had the culture medium removed and replaced with fresh medium containing 1000 μmol/L AAPH. The cells were then incubated at 37 °C in the dark for 6 h. For the positive control, the positive-control reagent was added to the wells, and the cells were incubated at 37 °C for 5 min. Subsequently, JC-1 reagent was added to all groups, and the cells were incubated at 37 °C for 20 min. After incubation, the supernatant was removed, and the cells were washed three times with PBS. Mitochondrial membrane potential was assessed under a laser-scanning confocal microscope (green fluorescence: Ex/Em = 510/527 nm; red fluorescence: Ex/Em = 585/590 nm), and fluorescence intensity was quantified using ImageJ.

#### 2.2.3. Ultrastructure of BMECs Observed Under TEM

Monolayer-cultured BMECs (≤70% confluence) were rinsed, and the medium was discarded. Trypsin was added to digest the cells, and when they detached, complete medium was added to stop the reaction. The cell monolayer was gently pipetted to lift the cells, and the suspension was transferred to a centrifuge tube. Cells were then pelleted by centrifugation at 3000 rpm for 3–5 min, yielding a pellet approximately the size of a mung bean. After discarding the supernatant, the pellet was resuspended in electron-microscopy fixative (pre-equilibrated to room temperature), dispersed by gentle pipetting, and fixed at room temperature in the dark for 30 min. The samples were then stored at 4 °C, transported on ice, and delivered to Servicebio Technology Co., Ltd. (Wuhan, China) for analysis.

#### 2.2.4. Immunofluorescence Staining of Oxidative Stress-Related Antibodies

Immunofluorescence staining was used to visualize the expression of oxidative stress-related antibodies in BMECs following AAPH exposure. BMECs were seeded onto glass coverslips. In the AAPH group, cells were exposed to AAPH for 6 h to induce oxidative stress. In the NAC group, cells were pretreated with AAPH for 6 h, followed by incubation with NAC to attenuate oxidative stress. The control group received PBS only. After treatment, cells were fixed with 4% paraformaldehyde for 15 min. The fixative was aspirated, and the coverslips were washed three times with PBS (5 min each). Permeabilization was performed using 0.1% Triton X-100 for 10 min, followed by three washes with PBS (5 min each). Primary antibodies were applied and incubated overnight at 4 °C in a humidified chamber. After incubation, coverslips were washed three times with PBS (5 min each) and then incubated with the corresponding secondary antibodies for 1 h at room temperature in the dark. After three additional washes with PBS (5 min each), nuclei were counterstained with DAPI for 10 min at room temperature in the dark. Detailed information on the antibodies used is as follows (Table 1). Slides were mounted with the antifade mounting medium. Images were acquired using a laser confocal microscope, and the mean fluorescence intensity of each antibody was quantified using ImageJ.

#### 2.2.5. Transcriptional Levels of Oxidative Stress-Related Genes in BMECs Detected by qPCR

Total RNA was extracted from BMECs using the AXYGEN kit, following the manufacturer’s instructions. Reverse transcription was performed using the PrimeScript RT Reagent Kit (Vazyme, Nanjing, China) at 37 °C for 15 min, followed by 85 °C for 5 s. RNA concentration and purity (A260/A280) were measured using a microplate reader. The acceptable concentration range was 500–1000 ng/μL, and the acceptable purity range was 1.8–2.0. Complementary DNA was stored at −80 °C. β-actin was used as the endogenous control gene. Quantitative PCR conditions were as follows: 50 °C for 2 min; 95 °C for 10 min; followed by 95 °C for 15 s; and 60 °C for 60 s. Reactions were prepared according to the TB^®^ Green protocol. Primers for *β-actin*, *AMPK*, *CYP1A1*, *HMOX-1*, *NOS*, *mTOR*, and *SOD* were designed and synthesized by Shengong Biological Co., Ltd. (Shanghai, China). The primer sequences are shown in Table 2.

#### 2.2.6. The Expression Levels of Oxidative Stress-Related Proteins in BMECs Were Detected by Western Blot

Total cellular proteins were extracted according to the instructions of the total protein extraction kit. Proteins were separated using 10% sodium dodecyl sulfate-polyacrylamide gel electrophoresis (SDS-PAGE) and transferred to nitrocellulose membranes. The membranes were blocked with 5% BSA at room temperature for 4 h and then incubated overnight at 4 °C with primary antibodies against β-actin, AMPK, CYP1A1, HMOX-1, NOS, mTOR, and SOD. After five washes with tris-buffered saline containing Tween-20, the membranes were incubated with HRP-conjugated goat anti-rabbit or goat anti-mouse IgG for 1 h at room temperature. Protein bands were visualized using enhanced chemiluminescence and quantified using ImageJ for grayscale analysis.

#### 2.2.7. Data Processing and Analysis

The 2^−^^ΔCt^ method was used to analyze the qPCR and Western blot data, calculate the relative expression levels of the target genes and proteins. GraphPad Prism 8 software was employed for statistical analysis and graphical presentation. Comparisons among groups were performed using one-way analysis of variance (one-way ANOVA), followed by pairwise comparisons of group means based on the test results. A significance level of * *p* < 0.05 was considered statistically significant, while ** *p* < 0.01 and *** *p* < 0.001 were considered highly significant. To ensure the reliability, stability, and reproducibility of the experimental results, each experiment was independently repeated three times, with three biological replicates per experiment. All data are presented as the mean ± standard deviation (Mean ± SD) to reflect the dispersion and variability of the data.

## 3. Results

### 3.1. The Optimal Active Concentration of AAPH

**Figure 1:**
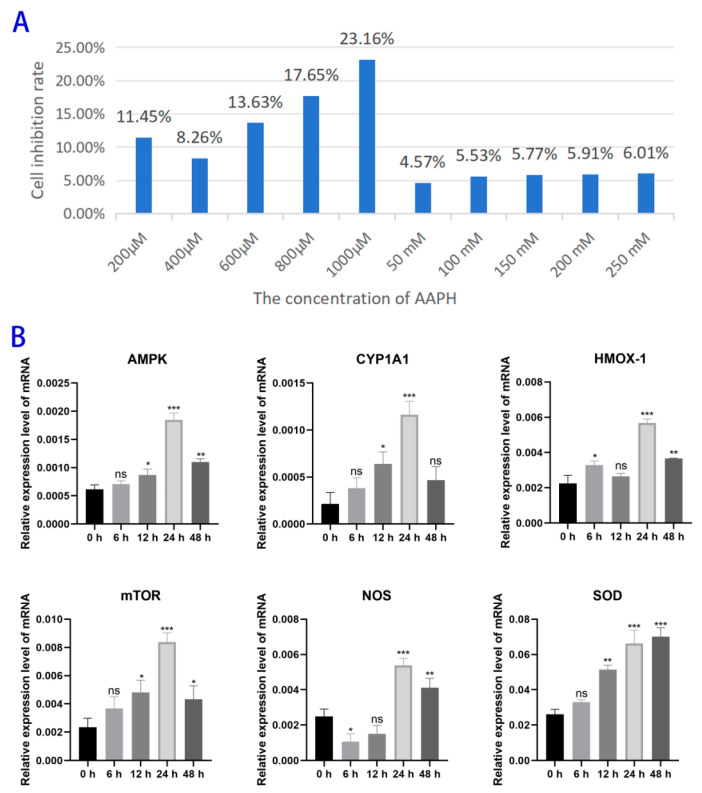
(**A**) Cell viability inhibition rate of BMECs treated with various concentrations of AAPH for 24 h, as determined by the CCK-8 assay. (**B**) Time course analysis of oxidative stress-related gene expression in BMECs treated with 1000 μM AAPH. Cells were harvested at 0, 6, 12, 24, and 48 h post-treatment, and the relative mRNA expression levels of AMPK, CYP1A1, HMOX-1, mTOR, NOS, and SOD were quantified by qPCR. The x-axis indicates the treatment duration (h). (ns: *p* > 0.05; * *p* < 0.05; ** *p* < 0.01; and *** *p* < 0.001).

The CCK-8 assay was used to measure the inhibition rate of cells treated with AAPH. The highest inhibition rate was observed when 1000 μM AAPH was added to the BMECs (Figure 1A). To determine the optimal treatment time, 1000 μM AAPH was added to BMECs, and total cellular RNA was extracted after 0, 6, 12, 24, and 48 h of treatment. The mRNA transcription levels of factors related to oxidative stress were measured at different time points by qPCR (Figure 1B).

### 3.2. Changes in Oxidative Stress-Related Indicators in BMECs After the AAPH Treatment

#### 3.2.1. ROS and Mitochondrial Membrane Potential in BMECs

**Figure 2:**
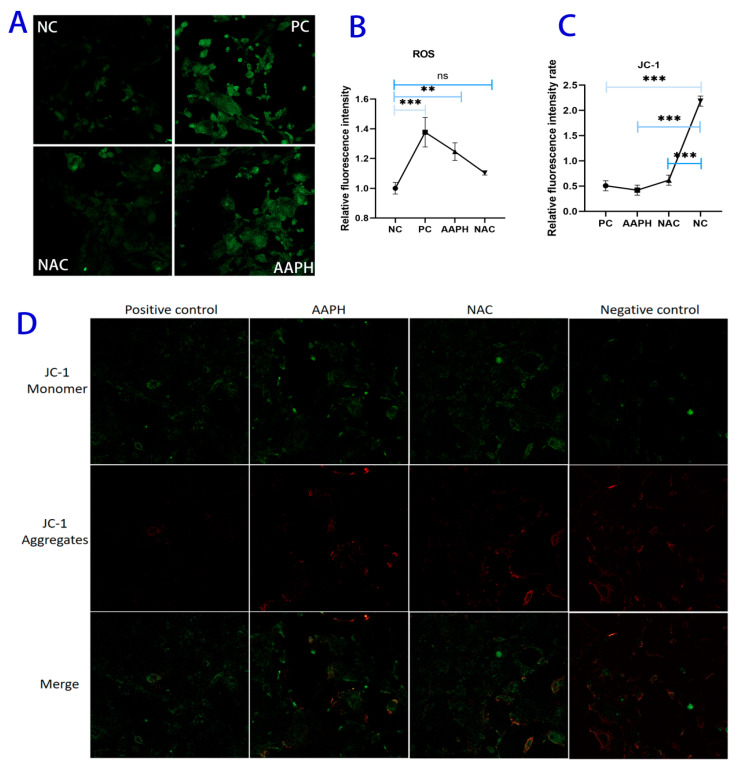
(**A**) Representative fluorescence images of intracellular ROS levels detected using the DCFH-DA probe. Green fluorescence intensity reflects the relative level of reactive oxygen species. Scale bar = 50 μm. (**B**) Quantitative analysis of relative ROS fluorescence intensity. Data are presented as mean ± SD from three independent experiments. NC: negative control (untreated cells); PC: positive control (cells treated with the ROS-positive control reagent provided in the kit); AAPH: cells treated with 1000 μmol/L AAPH for 6 h; NAC: cells pretreated with N-acetylcysteine prior to AAPH treatment. (**C**) Quantitative analysis of the relative ratio of red (J-aggregates) to green (JC-1 monomer) fluorescence intensity measured by the JC-1 assay. A decrease in this ratio indicates loss of mitochondrial membrane potential. (ns: *p* > 0.05; ** *p* < 0.01; and *** *p* < 0.001). (**D**) Representative fluorescence images of JC-1 staining. Red fluorescence indicates JC-1 aggregates in mitochondria with high membrane potential (healthy mitochondria), while green fluorescence indicates JC-1 monomers in mitochondria with low membrane potential (mitochondrial injury). The merged images show colocalization. Scale bar = 50 μm.

In the ROS assay, the DCFH-DA fluorescent probe was used. This probe enters cells and reacts with ROS to generate green fluorescence, with the fluorescence intensity positively correlating with intracellular reactive oxygen species levels—stronger green fluorescence indicates more severe oxidative stress. In the JC-1 mitochondrial membrane potential assay, the JC-1 probe exhibits different fluorescence states depending on changes in mitochondrial membrane potential: when the mitochondrial membrane potential is high, JC-1 aggregates to form J-aggregates that emit red fluorescence, indicating normal mitochondrial function; when the mitochondrial membrane potential decreases, JC-1 exists in monomeric form and emits green fluorescence, suggesting mitochondrial damage or early-stage apoptosis. Compared with the negative control (NC) group, the AAPH group and the positive control (PC) group showed a larger ROS-positive area in BMECs, accompanied by a significant increase in mean fluorescence intensity (*p* < 0.001). In the NAC group, the ROS-positive area was effectively reduced, and the mean fluorescence intensity showed no significant difference compared with the NC group (*p* < 0.05) (Figure 2A,B). Moreover, compared with the NC group, the PC, AAPH, and NAC groups exhibited weaker red fluorescence and stronger green fluorescence, reflecting a significant decrease in mitochondrial membrane potential (*p* < 0.001) (Figure 2C,D). They showed that, compared with the control group, the AAPH-treated group exhibited a significant increase in green fluorescence in the ROS assay, along with decreased red fluorescence and increased green fluorescence in the JC-1 assay, indicating that AAPH successfully induced oxidative stress and caused mitochondrial dysfunction. After NAC pretreatment, both fluorescence patterns recovered toward those of the control group, further confirming that NAC effectively alleviates AAPH-induced oxidative damage and mitochondrial injury.

#### 3.2.2. Transmission Electron Microscopy Results of BMECs 

**Figure 3:**
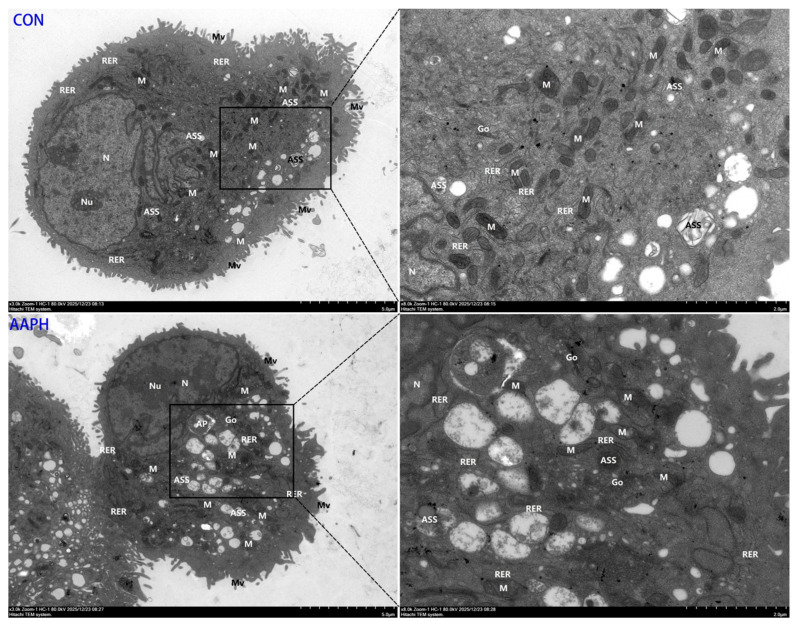
Transmission electron microscopy (TEM) analysis of ultrastructural morphology in BMECs. Representative TEM images showing the ultrastructure of BMECs in the control group (upper panels) and AAPH-treated group (lower panels). In the control group, cells display intact plasma membranes with abundant microvilli, uniformly distributed organelles, normal mitochondria with well-organized cristae, and orderly rough endoplasmic reticulum. In the AAPH-treated group, ultrastructural alterations include reduced microvilli, condensed cytoplasmic matrix with increased electron density, extensive vacuolization, mitochondrial swelling with cristae loss and matrix dissolution, and localized distortion of rough endoplasmic reticulum. Abbreviations: Mv, microvilli; N, nucleus; Nu, nucleolus; M, mitochondria; RER, rough endoplasmic reticulum; Go, Golgi apparatus; ASS, autophagolysosomes; AP, autophagosomes. Scale bar = 2 μm.

In the control group (Figure 3-CON), BMECs maintained a normal ultrastructural morphology. The cells appeared oval, with intact and continuous plasma membranes surrounded by abundant microvilli that were mostly uniform in thickness and evenly distributed. The cytoplasmic matrix was homogeneous, and organelles were evenly dispersed, with most retaining normal structural integrity. The nucleus was irregularly shaped but had an intact nuclear envelope. Euchromatin was uniformly distributed, and the nucleolus was compact and dense. Most mitochondria exhibited normal morphology, with intact membranes, a uniform matrix, and regularly arranged cristae. The rough endoplasmic reticulum showed no obvious dilation and was orderly arranged, with ribosomes clearly attached to its surface. The Golgi apparatus exhibited no evident hyperplasia or hypertrophy. Autophagolysosomes were present in relatively large numbers. In the AAPH group (Figure 3-AAPH), BMECs exhibited evident ultrastructural damage. The plasma membrane remained intact, but the number of microvilli was reduced, and some appeared significantly swollen and thickened. The cytoplasmic matrix was condensed and exhibited increased electron density, and many organelles were extensively vacuolated. The nucleus remained irregularly in shape, with increased heterochromatin. The nuclear envelope was intact, and the nucleolus remained dense. Some mitochondria were mildly swollen, with locally pale matrix and reduced cristae. Many mitochondria showed matrix dissolution and loss of cristae, resulting in vacuolated structures. The rough endoplasmic reticulum was locally distorted and fragmented. The Golgi apparatus showed no obvious hyperplasia or hypertrophy. Autophagosomes were occasionally observed, and autophagolysosomeswere present in considerable numbers.

#### 3.2.3. Immunofluorescence Results of BMECs

**Figure 4:**
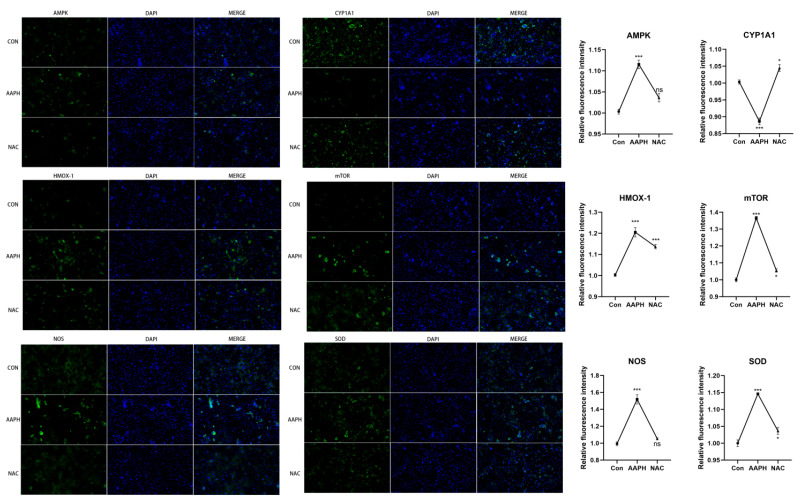
Immunofluorescence staining of oxidative stress-related proteins in BMECs. Representative immunofluorescence images showing the expression and subcellular localization of AMPK, CYP1A1, HMOX-1, mTOR, NOS, and SOD in BMECs under different treatment conditions. Cells were fixed, permeabilized, and incubated with specific primary antibodies followed by fluorescence-conjugated secondary antibodies. Nuclei were counterstained with DAPI (blue). Scale bar = 50 μm. Fluorescence intensities were quantified using ImageJ and are presented as mean ± SD from three independent experiments. NC: negative control (untreated cells); AAPH: cells treated with 1000 μmol/L AAPH for 6 h; NAC: cells pretreated with N-acetylcysteine prior to AAPH treatment. (ns: *p* > 0.05; * *p* < 0.05; and *** *p* < 0.001).

The expression levels of oxidative stress-related proteins in BMECs were assessed by immunofluorescence staining (Figure 4). Compared with the negative control (NC) group, the AAPH treatment group exhibited significantly higher fluorescence intensities of AMPK, HMOX-1, mTOR, NOS, and SOD (*** *p* < 0.001), while the fluorescence intensity of CYP1A1 was significantly decreased (*** *p* < 0.001). In the NAC pretreatment group, the fluorescence intensities of AMPK and NOS showed no significant difference compared with the NC group (*p* > 0.05). The fluorescence intensities of CYP1A1, mTOR, and SOD were significantly increased compared with the NC group (* *p* < 0.05), and the fluorescence intensity of HMOX-1 was also significantly elevated (*** *p* < 0.001). These results indicate that AAPH treatment modulates the expression of key oxidative stress-related proteins in BMECs, and NAC pretreatment exerts differential protective effects on these molecular targets.

#### 3.2.4. Changes in the Expression of Oxidative Stress-Related Genes in BMECs After AAPH Treatment

**Figure 5:**
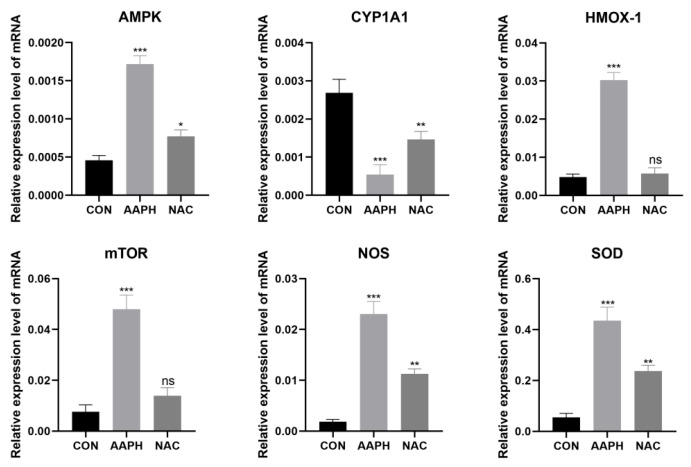
qPCR results for the mRNA expression levels of AMPK, CYP1A1, HMOX-1, mTOR, NOS, and SOD in BMECs across different treatment groups (ns: *p* > 0.05; * *p* < 0.05; ** *p* < 0.01; and *** *p* < 0.001).

Compared with the control group, the transcription level of AMPK mRNA was significantly higher in the AAPH group (*p* < 0.001). Although the level decreased in the NAC group, it remained higher than in the control group (*p* < 0.05). The transcription level of CYP1A1 mRNA was significantly lower in the AAPH group (*p* < 0.001), and although it slightly increased in the NAC group, it remained lower than in the control group (*p* < 0.01). The transcription levels of HMOX-1 and mTOR mRNA were significantly higher in the AAPH group (*p* < 0.001). In the NAC group, the levels of HMOX-1 and mTOR mRNA significantly decreased, with no significant difference compared to the control group (*p* > 0.05). The transcription levels of NOS and SOD mRNA were significantly higher in the AAPH group (*p* < 0.001). Although these levels decreased in the NAC group, they remained higher than in the control group (*p* < 0.01) (Figure 5).

#### 3.2.5. Changes in the Protein Expression of Oxidative Stress-Related Proteins in BMECs After AAPH Treatment

**Figure 6:**
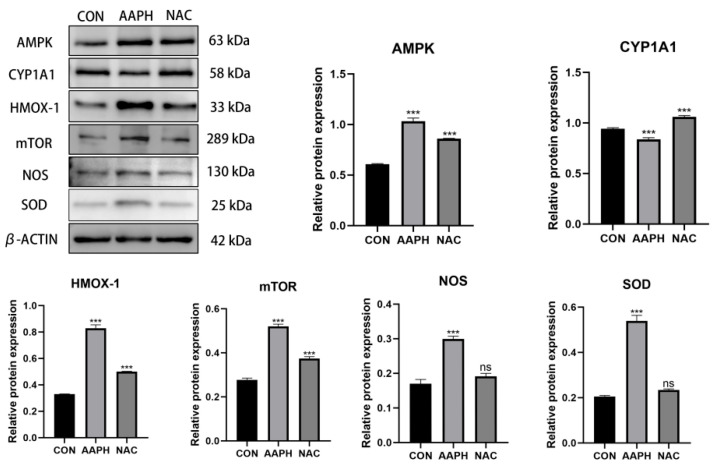
Western Blot results showing the protein expression of AMPK, CYP1A1, HMOX-1, mTOR, NOS, and SOD in BMECs across different treatment groups (ns: *p* > 0.05; *** *p* < 0.001).

Compared with the control group, the protein expression of AMPK was significantly higher in the AAPH group (*p* < 0.001). Although it decreased in the NAC group, it remained higher than in the control group (*p* < 0.001). The protein expression of CYP1A1 was significantly lower in the AAPH group (*p* < 0.001), but it significantly increased in the NAC group (*p* < 0.001). The protein expression of HMOX-1 and mTOR was significantly higher in the AAPH group (*p* < 0.001). In the NAC group, these levels decreased but remained higher than in the control group (*p* < 0.001). The protein expression of NOS and SOD was significantly higher in the AAPH group (*p* < 0.001). In the NAC group, both the expression of NOS and SOD significantly decreased, with no significant difference compared to the control group (*p* > 0.05) (Figure 6).

## 4. Discussion

In the present study, we established an in vitro oxidative stress model in BMECs using AAPH and characterized the associated cellular and molecular changes. The optimal treatment condition was determined as 1000 μmol/L AAPH for 6 h, based on cell viability and expression profiles of oxidative stress-related genes. Under these conditions, AAPH treatment significantly increased intracellular ROS levels, decreased mitochondrial membrane potential, and induced distinct ultrastructural alterations, including mitochondrial swelling, cristae loss, and vacuolization. These findings confirm that AAPH effectively induces oxidative stress and mitochondrial dysfunction in BMECs.

AAPH is a water-soluble azo compound that decomposes at 37 °C to generate carbon-centered radicals, which are rapidly converted to peroxyl radicals in the presence of oxygen. These peroxyl radicals initiate lipid peroxidation chain reactions, leading to the depletion of endogenous antioxidants and the accumulation of ROS [11]. Compared with H_2_O_2_, which decomposes rapidly and produces a transient oxidative burst, AAPH provides a sustained and controllable free radical flux, making it particularly suitable for modeling chronic oxidative stress conditions such as those occurring in the mammary gland of periparturient dairy cows [12]. While AAPH has been widely used in various cell types [13], its application in BMECs has not been previously reported. Our results demonstrate that AAPH-treated BMECs recapitulate key features of oxidative stress observed in vivo, including ROS overproduction, mitochondrial injury, and ultrastructural damage.

In this study, AAPH treatment significantly upregulated the expression of AMPK, HMOX-1, mTOR, NOS, and SOD, while downregulating CYP1A1 expression. These molecular changes collectively highlight the complex regulatory mechanisms underlying oxidative stress-induced damage in BMECs. The significant activation of AMPK indicates that the cells are experiencing energy stress. As a central regulator of cellular energy metabolism, AMPK promotes catabolism by phosphorylating downstream target proteins to maintain ATP levels [14,15]. However, the concurrent upregulation of mTOR in this study appears to contradict the classical view that AMPK-mediated inhibition of mTOR signaling occurs under energy stress. Further analysis suggests that this “AMPK-mTOR co-activation” may reflect the complexity of metabolic regulation under oxidative stress: AAPH-induced mitochondrial dysfunction triggers energy stress, activating AMPK to compensate for energy loss, the cell may retain basal mTOR activity through alternative pathways such as PI3K/Akt or stress-induced growth factor signaling, attempting to preserve anabolic processes essential for cell survival [16,17]. If this contradictory activation continues, it could create an imbalance between autophagy and protein synthesis, ultimately promoting cell apoptosis. The upregulation of HMOX-1, SOD, and NOS demonstrates a robust activation of the cellular antioxidant defense system. As a key antioxidant enzyme regulated by the Nrf2 pathway, the significant upregulation of HMOX-1 represents a classic protective response to oxidative damage. HMOX-1 exerts anti-inflammatory, antioxidant, and signal-regulating effects by catalyzing the degradation of heme into carbon monoxide, bilirubin, and free iron [18,19]. Although NAC pretreatment significantly reduced HMOX-1 expression, it failed to restore it to baseline levels, suggesting that AAPH-induced oxidative stress may have irreversibly altered cellular redox homeostasis to some extent. The upregulation of SOD aims to accelerate the removal of superoxide anions, but its byproduct, hydrogen peroxide, requires further metabolism by enzymes such as glutathione peroxidase. If not adequately processed, hydrogen peroxide can generate more toxic hydroxyl radicals through the Fenton reaction, creating an “antioxidant enzyme-mediated oxidation” paradox [20]. The upregulation of NOS indicates that oxidative stress may activate inflammation-related nitric oxide signaling pathways. The nitric oxide (NO) produced reacts with superoxide anions to form peroxynitrite, which can further exacerbate lipid peroxidation and protein nitration damage. The significant downregulation of CYP1A1 expression is an important finding. As a phase I drug-metabolizing enzyme and a classic target gene of the aryl hydrocarbon receptor (AhR), this downregulation may occur through multiple mechanisms: oxidative stress could inhibit AhR nuclear translocation or DNA binding; it may also represent an adaptive response where cells actively reduce metabolic burden during energy crisis; additionally, AAPH metabolites or lipid peroxidation products may downregulate CYP1A1 via competitive inhibition of the AhR signaling pathway [21]. The pathological significance of CYP1A1 downregulation includes reduced capacity for the metabolic clearance of endogenous or exogenous toxins, which may result in the accumulation of toxic substances. Furthermore, as CYP1A1 is involved in estrogen metabolism, its downregulation could disrupt endocrine homeostasis in mammary epithelial cells. NAC pretreatment significantly restored CYP1A1 expression, confirming that this change is oxidative stress-dependent. This finding supports the potential for antioxidant intervention to restore normal cellular metabolic function.

In addition, several limitations should be acknowledged. First, this study was conducted in an in vitro system using a single cell type (BMECs), which cannot fully replicate the complex in vivo microenvironment involving interactions among immune cells, stromal cells, and mammary tissue architecture. Therefore, the findings obtained in this model require further validation in animal models or clinical samples. Second, although the AAPH-induced oxidative stress model mimics chronic oxidative conditions, the controlled in vitro setting may not fully capture the dynamic metabolic, hormonal, and immunological fluctuations that occur in the mammary gland of periparturient dairy cows. Third, the use of a single treatment time point and concentration, while optimized, may not reflect the temporal dynamics of signaling pathway activation and cellular adaptation. Fourth, the molecular mechanisms proposed, particularly the paradoxical co-activation of AMPK and mTOR, warrant further investigation using specific inhibitors or genetic approaches to establish causality. Future studies should also explore the in vivo relevance of CYP1A1 downregulation and the potential of NAC as a therapeutic strategy in the context of bovine mastitis.

## 5. Conclusions

In summary, this study successfully established an AAPH-induced oxidative stress model in BMECs. The results suggest that AAPH treatment recapitulates several cellular events associated with oxidative stress, including energy metabolic reprogramming, activation of the antioxidant defense system, initiation of inflammatory signaling, and metabolic dysfunction. Notably, the observed co-activation of AMPK and mTOR, along with the downregulation of CYP1A1, may be involved in the cellular response to oxidative stress in vitro. However, as these findings are derived from an in vitro cell model, they cannot fully account for the complex interactions among metabolic, endocrine, and immune factors that occur in vivo during mastitis. Therefore, the proposed mechanisms should be considered as potential contributing pathways that warrant further validation in vivo. The protective effects of NAC in this model support its utility as a positive control, and the established platform may serve as a useful tool for preliminary screening of antioxidant compounds or for mechanistic studies under controlled oxidative conditions.

## Figures and Tables

**Table 1:** Detailed information about antibodies.

Antibody Name	Production Company	Product Item Number	Dilution Ratio
AMPK	Proteintech Group, Inc. (Wuhan, China)	10929-2-AP	1:50
CYP1A1	Affinity biosciences (Changzhou, China)	AF5312	1:50
HMOX-1	Proteintech Group, Inc.	10701-1-AP	1:50
NOS	Affinity biosciences	AF0199	1:50
mTOR	Cell Signaling Technology, Inc. (Danvers, MA, USA)	2972S	1:50
SOD	Affinity biosciences	AF5144	1:50
*β*-actin	Proteintech Group, Inc.	20536-1-AP	1:50

**Table 2:** Primer sequences for qPCR.

Gene Name	Primer Sequence (5′–3′)	TM (°C)	Gene Accession Number
*AMPK*	F: GTGGTGACCCTCAAGACCAG	58.3	NC_037330.1
	R: TTCCGGATGAGGTTTCAGG		
*CYP1A1*	F: TGCAGGAGAACATCCCTACC	56.7	NC_037348.1
	R: GGTAGGGTGATGAGGTCCAC		
*HMOX-1*	F: CTGACAGCATGCCCCAGGAT	55.1	NC_037332.1
	R: CTTCTCCTGGGCTCTCTCCT		
*NOS*	F: CCCCAGACAGCTTCTACCT	55.5	NC_037331.1
	R: TCCTTTGTTACTGCTTCACC		
*mTOR*	F: TGCGGTCACTCGTCGTCAG	60.6	NC_037343.1
	R: TGCCAGCCTGCCACTCTTG		
*SOD*	F: ATCCACTTCGAGGCAAAGGG	57.8	NC_037328.1
	R: GTGAGGACCTGCACTGGTAC		
*β-actin*	F: TCTGGCACCACACCTTCTACAAC	60.1	NC_037329.1
	R: GGACAGCACAGCCTGGAT		

## Data Availability

The original contributions presented in this study are included in the article. Further inquiries can be directed to the corresponding authors.

## Abbreviations

The following abbreviations are used in this manuscript:

ROS	Reactive oxygen species
NOX	NADPH oxidases
NAC	N-carbamylglutamate
EDTA	Ethylene diamine tetraacetic acid
DAB	Diaminobenzidine
AMPK	Adenosine 5′-monophosphate (AMP)-activated protein kinase
CYP1A1	Cytochrome P450 1A1
HMOX-1	Heme oxygenase 1
SOD	Superoxide dismutase
NOS	Nitric oxide synthase
mTOR	Mammalian target of rapamycin
FBS	Fetal bovine serum
BSA	Bovine serum albumin
HRP	Horseradish peroxidase
